# Supplementary material for: Cerebral malaria is associated with differential cytoadherence to brain endothelial cells
Source: EMBO Mol Med. 2019 Jan 4;11(2):e9164. doi: 10.15252/emmm.201809164 (PMC6365927; doi:10.15252/emmm.201809164)
Supplement: Supplementary file 1 — Appendix [file EMMM-11-e9164-s001.pdf]

Appendix

Cerebral malaria is associated with differential cytoadherence to brain endothelial cells.

Table of contents

Appendix Table S1. In silico characterisation of *var* gene primers.

Appendix Table S2. Correlation between binding of the clinical isolates to HBMEC or HDMEC and transcript levels of *var* gene domains.

Appendix Figure S1. Characterisation of HBMEC and HDMEC and expression of ICAM-1, EPCR and CD36 at number of passages.

Appendix Figure S2. Cytoadherence of IT4var19 and three patient isolates to HBMEC in presence of human serum.

Appendix Figure S3. Inhibition of cytoadherence of IE from CM and UM cases to HBMEC and HDMEC by combined αICAM-1 antibody and rEPCR.

Primer name (Target domain type)	Forward primers 5'-3'<br><br>Mixed to a final total primer concentration of 20 µM	Reverse primers 5'-3'<br><br>Mixed to a final total primer concentration of 20 µM	Amplicon size<br><br>Median (Min - Max) bp	Coverage<br><br>Number of sequences amplified with intended domain class / number of sequences with intended domain class (%)	Specificity<br><br>Number of sequences amplified with intended domain class / number of domain sequences amplified (%)	Specificity graphic
CIDRa1.2-K (CIDRα1.2)	AAGGGATACTATTAAGTGGAATGA	YTTTGTCWAGTTTATCCATAACTTT	245 (245-245)	133/133 (100%) [133/138 (96%) of all CIDRα1.2]	133/133 (100%)	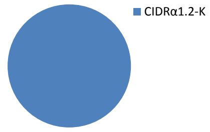
↑ Storm et al.						
CIDRa1.3-K (CIDRα1.3)	GTGGGAAAACGAAATTAAGGATTGT	TTTTGCTTCTCCTTGGTCAAGTTC	244 (244-244)	65/65 (100%) [65/80 (81%) of all CIDRα1.3]	65/65 (100%)	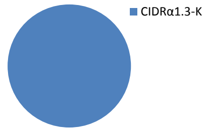
↑ Storm et al.						
VAR1-K	**Comment:**<br>**A summarised transcript level for VAR1 domains (CIDRα1.2+CIDRα1.3 K variants of a EPCR interacting residue corresponding to Q657 in HB3var03 - considered incompatible with binding). There is no overlap between targeted sequences. Primers can be used in multiplex. CIDRa1.2-K can serve as a specificity control for the CIDRa1.7 primer (that primer targets 41/78 (53%) of [illegible]**		245 (244-245)	198/198 (100%) [198/218 (91%) of all CIDRα1.2/1.3]	198/201 (99%)	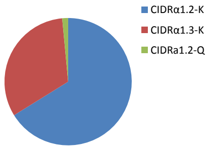
CIDRa1.1 (CIDRα1.1)	TGGGAACATCAACTTAAGGATTGCATA<br>TGGGAACATCAACTTAAGAATTGCATA<br>TGGGAACATGAACTTAAGGATTGCATA	TAAATCTTYCNTAAATTGATHCCAT	270 (270-273)	274/290 (95%)	274/277 (99%)	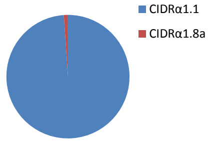
CIDRa1.8a (CIDRα1.8a)	ATAATTGTGAAATGAAAGGTTCA	TATGCAMTTCTTAAGTTTGGTTTCC	140 (140-140)	104/104 (100%)	104/104 (100%)	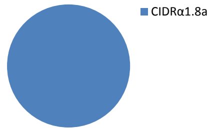
CIDRa1.8b (CIDRα1.8b)	AATAGACAGTATAATGTGGGAA<br>AAAGGATACTATAAAGTGGGAA	CAAAACATWTACAATTTTCGTTACAK	101 (98-101)	61/61 (100%)	61/102 (60%)<br>[All non-CIDRα1.8b targets have 2 mismatches to at least one primer. 56% of CIDRα1.4 off targets also targeted by CIDRa1.4/6a primer]	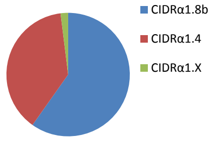
↑ Storm et al.		↑ Updated from Mkumbaye et al.. 3' "K" added for improved specificity				
CIDRa1.8 (CIDRα1.8)	Comment:<br>A summarised transcript level for CIDRα1.8 domains (CIDRα1.8a+8b). There is no overlap between targeted sequences.					
CIDRa1.DC8	Comment:<br>A summarised transcript level for DC8 domains (CIDRa1.1+CIDRα1.8a+8b). There is no overlap between targeted sequences.					
CIDRa1.4/6a (CIDRα1.4/6a)	AACTATCAAAAATGGAATGCTATTA<br>AACTATGAACAATGGAATGCTATTA<br>AACTATCAAAAATGGAATTGCTATTA<br>AACTATGAAAACTGGCAATGCTATTA<br>AACAATCAAATATGGAAATGCTATTA	AATGGGACAAACAAAAAACAAAATATG	163 (163-172)	CIDRα1.4: 281/345 (81%)<br>CIDRα1.6a: 31/54 (58%)	312/319 (98%)	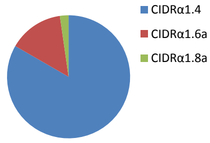
CIDRa1.5a (CIDRα1.5a)	GATTTATGGATTAAGAATTTATTAAG<br>GATTTGTGGGTTACGAATTTATTAAG<br>GATTTGTGGGTTACATATTTATTAAG	TAATTCATCCGTAAATTTCTTCCA<br>CAAATCTTCCTTAAGTTTTTTCCA<br>TAATTCATCCGTAAATTGATTCCA<br>CAAATCTTCTTTAAGTTTTTTCCA	306 (306-306)	98/104 (94%)	98/98 (100%)	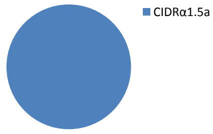

Primer name<br>(Target domain type)	Forward primers<br>5'-3'<br><br>Mixed to a final total primer concentration of 20 µM	Reverse primers<br>5'-3'<br><br>Mixed to a final total primer concentration of 20 µM	Amplicon size<br><br>Median (Min - Max)<br>bp	Coverage<br><br>Number of sequences amplified with intended domain class / number of sequences with intended domain class (%)	Specificity<br><br>Number of sequences amplified with intended domain class / number of domain sequences amplified (%)	Specificity graphic
CIDRa1.5b<br>(CIDRα1.5b)	ACGATACTATAGACTGGAAATACG<br>ATTGGGAAWATAAACTTAAGACCTG<br>TGGATACTACAGATTGGGATCGTA	AACCCATTGTTCAAAACATTTACA<br>AACCCATTTATCAAAACACGTACA<br>AACCCATTTATCAAAACACATACA	110 (98-113)	119/124 (96%)	114/114 (100%)	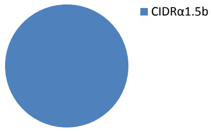
CIDRa1.5	Comment:<br>A summarised transcript level for CIDRα1.5 (CIDRa1.5a+CIDRa1.5b). There is no overlap between targeted sequences.					
CIDRa1.6a<br>(CIDRα1.6a)	AAACTATCAAATATGGCAATGCT<br>AAACTATGAAAAATGGCAATGCT	CAATTTGTAAGTTCGTTTTTCCA<br>ATTCGTAAGATCGTTCTTCCA	180 (178-181)	54/54 (100%)	54/56 (96%)	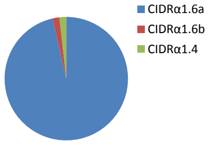
↑ Storm et al.						
CIDRa1.6b<br>(CIDRα1.6b)	ATAATACTAATGTSACGGATTGT	CAGTTTCTTTATACTATCCCATTC<br>ACATCCTTTATACTACCCCATTCC<br>AATTCCTTTATACTCTTCCATTCTG	107 (104-107)	81/81 (100%)	81/112 (72%)<br>[28% of the targeted genes are also targeted by two other primer sets: CIDRa1.4/6a and -1.7]	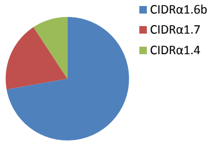
CIDRa1.7<br>(CIDRα1.7)	CGGAAACTATAACGTGGAACGATAA<br>CGGAAACTATAAGGTGGAACGATAA<br>CGGAAACTATAACGTGGAAAGATAA<br>GGATACTATAATGTGGAATGATAAA	TAGTTTCTTTATACTATTCCATTC<br>TAGTTCCTTTATACTATTCCATTC<br>TAGTTTCTTTATATTATTCCATTC<br>TAGTTTCTTTATACTACTCCATTC<br>TAATTCCTTTATATTATTCCATTC	148 (145-149)	277/282 (99%)	277/395 (70%)<br>[unintended targets are all *var1* CIDRα1.2]	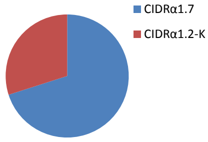
CIDRa1.A<br>(CIDRα1 of group A)	Comment:<br>A summarised transcript level for all group A CIDRα1 domains (CIDRa1.4-7 primers). There is an estimated 2% of genes targeted twice by these primer sets.					
CIDRa1.all<br>(All CIDRα1)	Comment:<br>A summarised transcript level for all CIDRα1 domain primers. There is an estimated 5% of genes targeted twice by these primer sets.					
CIDRd<br>(N-terminal CIDRδ)	TAAATGTAACTTAGATGTATGTGAAC<br>TAAATGTAACTTACATGTATGTGAAC<br>TAAATGTAACTTAGACGTATGTGAAC<br>TAAATGTTACTTAGATGTATGTGAAC<br>TAAATGTAACTTAGATATATGTGAAC<br>TAAATGTAAGTTAGATGTATGTGAAC	AATACTTTAACCAACGTTTAATCAATAC<br>AATACTTTAACCAACGCTTAATCAATAC<br>AATACTGCAACCAACGTTTAATCAATAC<br>AATGCTCTAACCAACGTTTAATGAATAC<br>AATACATCAACCAACGCTTAATCAATAC	104 (104-104)	308/410 (75%)	308/350 (88%)	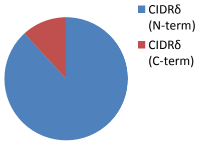
CIDRg3.1<br>(CIDRγ3.1)	TATGTATATGCTGATGAACGTATTAC	TTCTATCCATTTTTCTAAACATTC	174 (174-174)	81/81 (100%)	81/81 (100%)<br>[The N-terminal group A CIDRγ domains (ie. CIDRγ3 domains) are diversified into several small subgroups of which the CIDRγ3.1 is the largest (~20% of CIDRγ3)	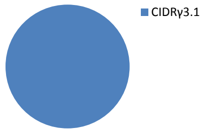
CIDRa3.1/2<br>(CIDRα3.1/2)	AHWWVCAAAAGACRTWCHATRATTT<br>AHWWVCAAAAGACRTTCAATCCT<br>ARAAAGTAAAGGATTATGTWGRTTT	TTTTTGTTCTCCAATRTATRGAATC	80 (80-81)	1479/1970 (75%)<br><br>CIDRα2-6: 1536/9142 (17%)	1479/1536 (96%)<br><br>CIDRα2-6: 1536/1536 (100%)	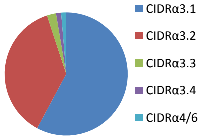

Primer name (Target domain type)	Forward primers 5'-3' Mixed to a final total primer concentration of 20 µM	Reverse primers 5'-3' Mixed to a final total primer concentration of 20 µM	Amplicon size Median (Min - Max) bp	Coverage Number of sequences amplified with intended domain class / number of sequences with intended domain class (%)	Specificity Number of sequences amplified with intended domain class / number of domain sequences amplified (%)	Specificity graphic
DBLa2/1.1/2/4/7 (DBLα2/1.1/2/4/7)	GATTAYGTBCCTCAATTTTTAMGWTGGT	TACAATCATATCCATTAWGACTACAA<br>TCACAATCGCATCCATTATGACTACAA	145 (145-157)	1422/1736 (82%)	1422/1489 (82%)	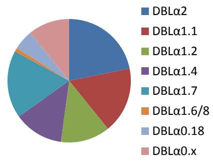
DBLa1.5/6/8 (DBLα1.5/6/8)	GATTAYGTBCCTCAATTTTTAMGWTGGT	TTTTAGTACAATCATAACCATCACCA<br>GATTTGTTTTWTTACAATCGTAACCCTC<br>ACAATCCTCACCATCACCACTACAAT<br>CGTGATATATCTGTTTKAGTACAATC<br>GATCTGTTCGTTTACAATCGTAACCCTC	157 (144-173)	596/812 (73%)	596/666 (90%)	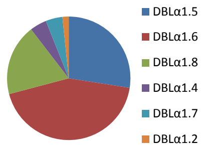
DBLa1all (DBLα1.1-8)	TTGGGAAATGTRTTRGTTACAGCAAA<br>TTGGGAAATGTGTTAGTTATGGCAAA<br>TTGGGGAATTTGTTAGTTATGGCAAG<br>TTGGGGAACCTATTAGTTATGGCAA<br>TTAGGAAATATATTGGTAGCAGCAA<br>TTAGGAAATATCTTGGTCACAGCAA	CCTATATCNGCAAAACTKCKWGC	116 (116-128)	2211/2404 (92%)	2211/2211 (100%)	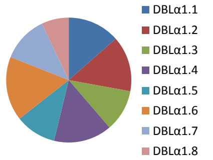
DBLb1/3-1 (Group A DBLβ1/3, subdomain 3)	TATACAAASAAGCAGAAATTTATGC<br>TACGCAAAAGCACGAATTGTTGCTA	TTATAATACCCAGGACCRCCATT	52 (50-52)	N/A (8% of DBLβ3) (25% of DBLβ1 not *var1*) [95% of ICAM1 binding group A DBLβ defined in Lennartz et al. 2017]	N/A	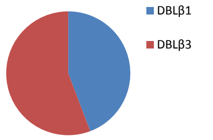
DBLb1/3-2 (Group A DBLβ1/3, subdomain 3)	AACATGCACKAGTCGATATTGCT	CAGTAGAAGTWTTARGACCACCAT	51 (51-51)	N/A (5% of DBLβ3) (18% of DBLβ1 not *var1*)	N/A	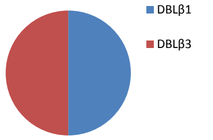
DBLb5-S1 (DBLβ5 subdomain 1)	AACAAAGTAGCGTATCAAATGCAT<br>AACAAARYRGCACAMCWAATGCATVA	CCTTKTGWTGCATYRSCCYTYAA	92 (89-95)	521/1428 (37%)	521/661 (79%) [No target overlap with other DBLβ primers)	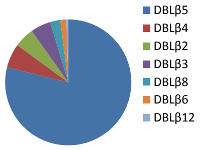
DC5 (DC5 DBLβ domain)	GTTGCTCCYMCTTTTTGTAATGT<br>CCCCCHCCYTTTTGTAAYGTNCC<br>TTGCACCCATTTTTTGTAATATGCC	ACCACRTTGGTCGCATCTTTGTC<br>CACTCACTATGTTGGTGYCATTTTT<br>GCCASCACTTCWACTCCNACCAC<br>YACAYTWACCACATTNGTSGM	69 (62-83)	388/500 (78%)	388/388 (100%) [No target overlap with other DBLβ primers)	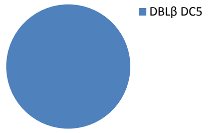
DBLe2 (DBLε2)	AAAWTTAATWGGTTTGGRAGCAC<br>AATTTRATWGGTTTAAATGCAYACA	GACATAATTGTTGYACTCTAGGAGRMA	86 (85-89)	335/416 (80%)	335/335 (100%)	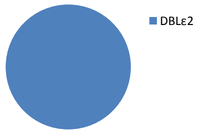
DBLe6 (DBLε6)	GTTGCATAYAATGAAGGTTATTTCCT<br>AAAACTRRACTTATGAATTSTGCCTACA<br>ATTTCTTCAATATGYCTAYACTSAAGGA	ATCHGTWCCTTTAACTATATCTCCATA<br>ATCTYTACCTTTTACTATATCAGCAAT	127 (117-135)	335/442 (76%)	335/387 (87%)	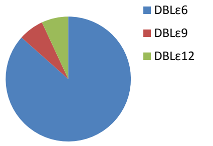
DBLe11 (DBLε11)	AAAGHATTACAAAAAGAYGCATAT<br>CATTACAAATATGTGCATATAATSAAG<br>AAATACAGGAATCAGCATACAACGAAG	TTCCTTTAATTAAATYASCATAATCWGCA<br>TCAGCATAACTYCTTTTCATATTTTCA	177 (151-178)	287/315 (91%)	287/287 (100%)	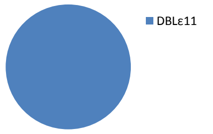

Primer name (Target domain type)	Forward primers 5'-3'<br><br>Mixed to a final total primer concentration of 20 µM	Reverse primers 5'-3'<br><br>Mixed to a final total primer concentration of 20 µM	Amplicon size<br><br>Median (Min - Max) bp	Coverage<br><br>Number of sequences amplified with intended domain class / number of sequences with intended domain class (%)	Specificity<br><br>Number of sequences amplified with intended domain class / number of domain sequences amplified (%)	Specificity graphic
DBLe13 (DBLε13)	CTGTTGCTGCAAATSAAGGATATAAT<br>CTGCTGYTGCAAATGATGCATATAAT	ATAATCRTAAAAACTRTATTTCAATGCAT	107 (107-107)	185/196 (85%)	185/185 (100%)	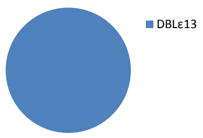
DBLe14 (DBLε14)	TTTAAYCAAGGAATWCTTTTAGGAA	ATATCTSCATARTCAGCAAAACTAT	95 (95-101)	117/137 (85%)	117/193 (61%)	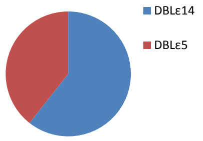
DBLe_all (DBLε2/6/11/13/14)	Comment:<br>A summarised transcript level for all DBLe primers. There is no overlap between the primer sets summarised.					
DBLz2a (DBLζ2)	CCTCAACGTTTRAGATGGATGAAG	ATTTTGCAAACATAYTCTCCCCATT	50 (50-50)	64/336 (19%)	64/142 (45%)	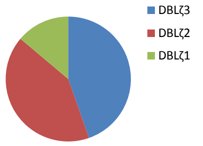
DBLz2b (DBLζ2)	AGTGACCCTCCTGTGGATGATTA	TGCAATAATTTTCACTCCATTCCTT	73 (73-73)	114/336 (34%)	114/114 (100%)	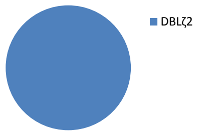
DBLz2c (DBLζ2)	ATGTRCCTCAAATACTTAGATGGATWA	TTCTTGCAATTCACAAAAATGTTC	62 (62-62)	104/336 (31%)	104/104 (100%)	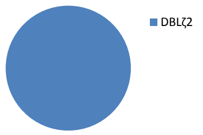
DBLz2 (DBLζ2)	Comment:<br>A summarised transcript level for DBLζ2 domains (DBLz2a+DBLz2b+DBLz2c). There is no overlap between the primer sets summarised.			282/336 (84%)	282/371 (76%)	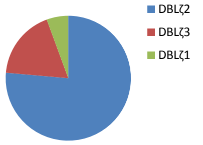
DBLz3 (DBLζ3)	AACCTCCTTATGTTGATTACATTCCACA<br>ATCCTCCTTATGATGATTATATWCCWCA	TTCTGACCATTCAGTCATCCATCT	59 (59-59)	90/264 (34%)	90/90 (100%)	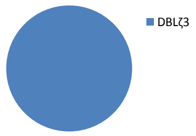
DBLz5 (DBLζ5)	GATTATGATTATATYCCTCAACCTT	TTCRCYCCATTCACTTAKCCAKCG	51 (51-51)	233/248 (94%)	233/233 (100%)	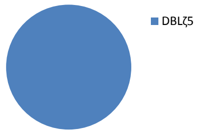
DBLz4 (DBLζ4)	AACCTCCTGATTATGATTATATACCT	CAATAATATTCACTCCATTCTTGC	67 (67-67)	281/302 (93%)	281/390 (72%)<br>[55% of targets also targeted by DBLz6]	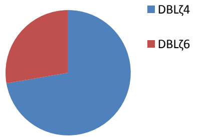

Primer name (Target domain type)	Forward primers 5'-3' Mixed to a final total primer concentration of 20 µM	Reverse primers 5'-3' Mixed to a final total primer concentration of 20 µM	Amplicon size Median (Min - Max) bp	Coverage Number of sequences amplified with intended domain class / number of sequences with intended domain class (%)	Specificity Number of sequences amplified with intended domain class / number of domain sequences amplified (%)	Specificity graphic
DBLz6 (DBLζ6)	CCTGATTATGATTATATWCCYCAACCTT	TTCACTCCATTCTTGCATSMAACG	54 (54-54)	324/400 (81%)	324/444 (73%)	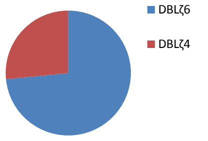
DBLz4/6 (DBLζ4/6)	Comment: Due to the considerable overlap between DBLz4 and DBLz6 primer sets, the transcript level for these domains were analysed using only the data from the primer reporting highest transcript level, *i.e* . either DBLz4 or the DBLz6 primer.					
DBLz_all (DBLζ)	Comment: The summarised transcript level for all DBLζ domains calculated as sum of DBLz2+z3+z4/6. There is no overlap between the primer sets summarised.					
var3 (DBLε8 of DC3 aka. DBLζ3-DBLε8)	AAGAGGATCTACTTAATGCTGCTTTTAG	AACTGAACTTCATAGCCTCATATGC	99 (99-99)	164/164 (100%)	164/164 (100%)	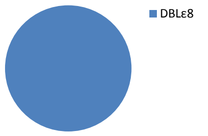
↑ "DBLe8" from Lavstsen et al. PNAS, 2012						
var2csa (DBLpam3 aka. DBL3x)	AATGGGACAAACAAAAAACAAAATATG	GCTGATATACATTCAGGATAATTTTC	94 (94-94)	265/265 (100%)	265/265 (100%)	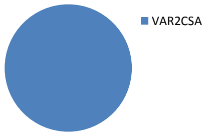
↑ "T12/T13" from Sander et al. PLoS One, 2009						

Appendix Table S1. In silico characterisation of *var* gene primers.

Sequences and predicted coverage and specificity of the primer sets, with the targeted domains in a pie chart. Primers are described in detail by Mkumbaye et al and 4 additional primer sets used in our study are indicated with an arrow.

**Target**		**Predicted Receptor**	**Basal binding**	**ICAM-1 dependent binding**		**EPCR dependent binding**		**CD36 dependent binding**	
**Domain**	**Group**		**HBMEC**	**HBMEC**	**HBMEC**	**HBMEC**	**HDMEC**	**HDMEC**	**HDMEC**
			**CM**	**CM**	**UM**	**CM**	**CM**	**CM**	**UM**
**CIDRα1.DC8**	B/A	EPCR			-0.60 (18)	0.59 (11)			
**CIDRα1.A**	A	EPCR				0.61 (11)			
**CIDRα1_all**	A & B/A	EPCR			-0.58 (17)	0.54 (10)			
DBLζ4 or DBLζ6						0.72 (11)			
DBLζ5								0.58 (11)	
**DBLζ_all**			-0.52 (16)	-0.65 (13)					
DBLε6				-0.71 (13)					
**DBLε_all**				-0.63 (13)					0.50 (18)
DC5	A	PECAM-1		0.57 (15)					
DBLα1ALL	A						0.71 (9)		
DBLα2/1.1/1.2/1.4/1.7	A					0.59 (10)			

Appendix Table S2. Correlation between binding of the clinical isolates to HBMEC or HDMEC and transcript levels of *var* gene domains.

The correlation coefficient r and P-value were calculated by the two-tailed non-parametric Spearman test for binding of the clinical isolates to HBMEC or HDMEC and the Tu values of the primer sets. The observations are not corrected for multiple comparisons. The correlation coefficient is shown for basal binding (in absence of inhibitors) and percentage inhibition by rEPCR, αICAM-1 or αCD36 antibody. Group sizes of correlation data are in brackets. The target domains are shown in the first column with specific domain groups in bold. In the matrix, the r-values are only shown when the P-value < 0.1 and $r \geq 0.5$ and when at least 25% of the isolates in each group have a Tu value of ≥ 16. The colour indicates the range of the P value; yellow: $0.05 > P < 0.1$, green: $0.01 > P < 0.05$ and red: $P < 0.01$. Note that there are positive and negative correlations.

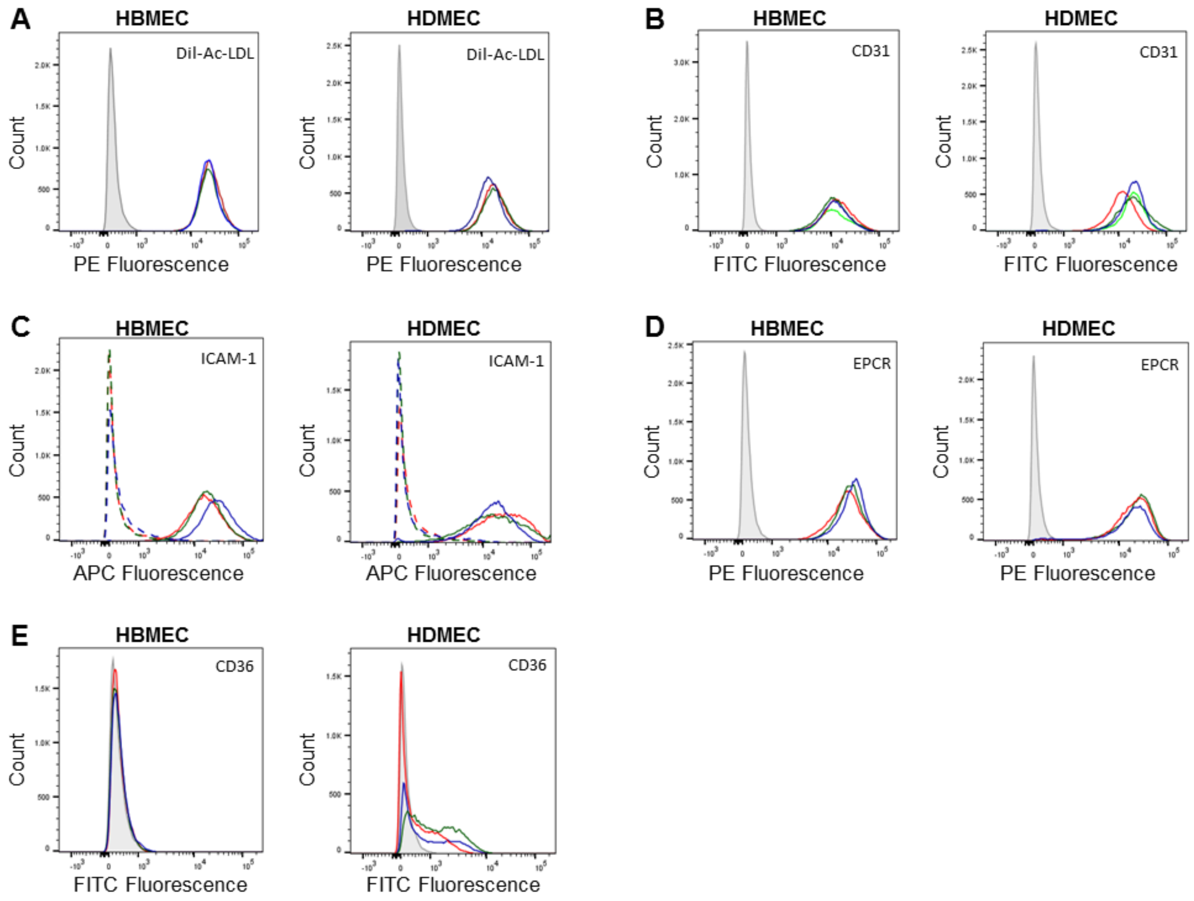

Appendix Figure S1. Characterisation of HBMEC and HDMEC and expression of ICAM-1, EPCR and CD36 at number of passages.

A Characterisation of HBMEC and HDEMEC by the uptake of Dil-labelled acetylated low density lipoprotein as measured by flow cytometry. Unlabelled cells are depicted in grey and labelled cells in colour.

B Levels of CD31 expression on HBMEC and HDMEC was measured by flow cytometry. Unlabelled cells are depicted in grey and labelled cells in colour.

C Upregulation of ICAM-1 by 10 ng/ml TNF on HBMEC and HDMEC, measured by flow cytometry. The dotted lines represent unstimulated labelled cells and the continuous lines represent the TNF-stimulated labelled cells.

D Levels of EPCR expression on TNF-stimulated HBMEC and HDMEC was measured by flow cytometry. Unlabelled cells are depicted in grey and labelled cells in colour.

E Levels of CD36 expression on TNF-stimulated HBMEC and HDMEC was measured by flow cytometry. Unlabelled cells are depicted in grey and labelled cells in colour.

Shown are representative flow cytometry experiments. HBMEC: blue = passage 5, light green = passage 7 (only in B), red = passage 8 and dark green = passage 9. HDMEC: blue = passage 4, red = passage 6 and green = passage 8.

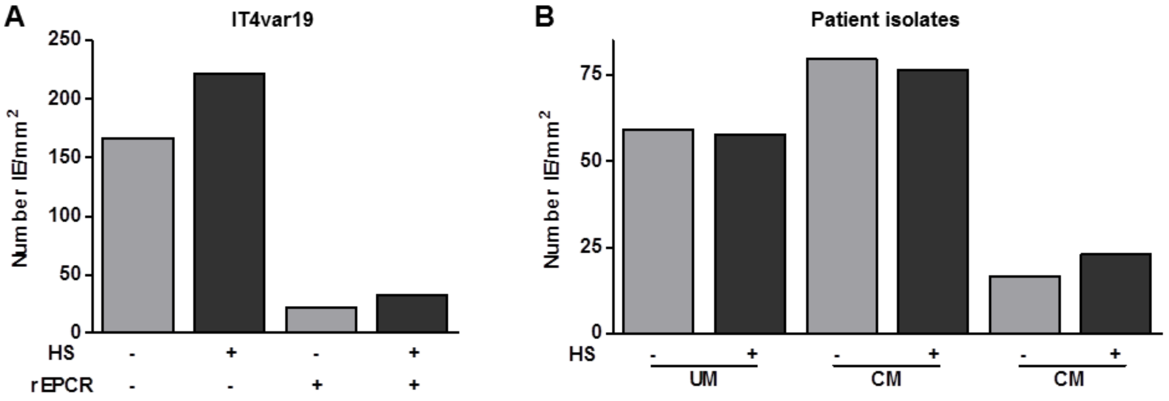

Appendix Figure S2. Cytoadherence of IT4var19 and three patient isolates to HBMEC in presence of human serum.

A Binding of the PfEMP1-DC8 variant IT4var19 to HBMEC was determined under flow conditions without and with 10% human serum (HS) and in the absence and presence of 50 µg/ml rEPCR. Number of IE bound per mm^2 EC surface was calculated.

B Binding of 1 UM and 2 CM isolates, which were shown to be mainly EPCR-dependent binders, to HBMEC under flow conditions without and with 10% human serum (HS). Number of IE bound per mm^2 EC surface was calculated.

Data information: shown are single representative experiments and thus statistical differences cannot be determined.

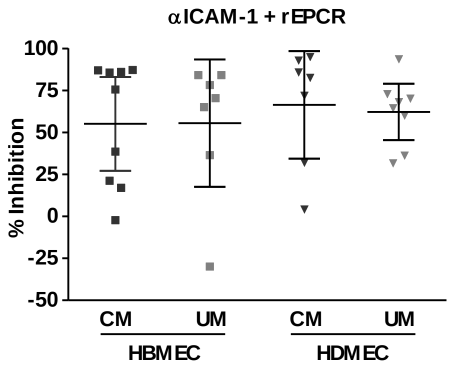

Appendix Figure S3. Inhibition of cytoadherence of IE from CM and UM cases to HBMEC and HDMEC by combined αICAM-1 antibody and rEPCR.

IE were isolated and binding to HBMEC and HDMEC was determined under flow conditions in the presence of 5 µg/ml αICAM-1 antibody and 50 µg/ml rEPCR. Number of IE bound per mm^2 EC surface was measured and percentage inhibition was calculated relatively to binding in the absence of inhibitors. Shown are the mean ± 95% CI and no significant differences were determined with a two-tailed unpaired t-test.
